# Preoperative weight loss by noninvasive approach in patients with obesity scheduled for bariatric and metabolic surgery: an update narrative review of indications and results available until 2024

**DOI:** 10.1007/s13304-025-02198-x

**Published:** 2025-04-12

**Authors:** Ilenia Grandone, Monica Nannipieri, Caterina Conte, Edda Cava, Luigi Schiavo

**Affiliations:** 1 Unit of Diabetology, Dietetics and Clinical Nutrition, Santa Maria Hospital, Terni, Italy; 2 Department of Clinical and Experimental Medicine, University of Pisa, Pisa, Italy; 3 San Raffaele Roma Open University, IRCCS MultiMedica, Milan, Italy; 4 Clinical Nutrition and Dietetics, San Camillo Forlanini Hospital, Rome, Italy; 5 Department of Medicine, Surgery and Dentistry, University of Salerno, Baronissi, Italy

**Keywords:** Metabolic and bariatric surgery, Preoperative weight loss, Anti-obesity drugs, Space occuping devices

## Abstract

Metabolic and bariatric surgery (MBS) is the most effective treatment for severe obesity and its metabolic complications. Currently, most MBSs are performed laparoscopically. However, high weight associated with an enlarged liver (especially the left lobe liver section, LLLS) may complicate the technical aspects of this surgery. Therefore, before MBS, moderate preoperative weight loss (PreopWL), and reduction in LLLS are desirable. Moreover, studies are inconclusive regarding which is the best approach to apply. This narrative review aimed to describe the current scientific evidence on the effect of a noninvasive approach, such as dietary or pharmacotherapy or space-occupying devices on PreopWL, peri-operative complications, hospital length of stay, and post-operative complications in patients with obesity scheduled for MBS. We conducted a literature search and screening for relevant publications from January 2010 to June 2024. We found that PreopWL before MBS is helpful for both patients and surgeons, as it leads to various benefits, such as a decrease in body weight and LLLS size, a lower risk of intra- and post-operative complications, shorter surgery times, and reduced hospital stays. In this context, concerning dietary approaches, several dietary protocols have been introduced over time, among which very low-calorie diets and very low energy ketogenic therapy are widely prescribed; however, larger randomized-controlled trials (RCTs) with well-defined dietary protocols are necessary to make definitive conclusions. Obesity management medications, such as the lipase inhibitor orlistat, phentermine/topiramate, naltrexone/bupropion, the glucagon-like peptide-1 receptor agonists (GLP-1RAs) liraglutide and semaglutide, and the novel dual glucose dependent insulinotropic peptide (GIP)/GLP-1 receptor agonist tirzepatide, has shown to be effective in promoting PreopWL before MBS; however, larger, well-designed RCTs are needed to establish optimal treatment protocols and assess their true benefits in patients scheduled for MBS. Space-occupying devices such as the swallowable intragastric balloon and hydrogel capsules, represent a promising tools but further research is essential to confirm their role.

## Introduction

Metabolic and bariatric surgery (MBS) has been shown to be safe and remain the most effective and durable treatment for clinically severe obesity, with a documented reduction in all-cause mortality and long-term survival benefits [1]. In 2011, the American Society for Metabolic and Bariatric Surgery (ASMBS) published a position statement on preoperative weight loss requirements [2]. The summary and recommendations from the 2011 statement concluded that there were no Level I studies or evidence-based reports that documented any benefit or need for a 3–18 month preoperative dietary weight loss program before MBS. Preoperative weight loss was found to be unsupported by any degree of medical evidence and, therefore, inappropriate, counterproductive, and potentially harmful due to unnecessary delays and interference with potentially life-saving treatment. Preoperative weight loss has previously been studied as a predictive factor for the success of outcomes after MBS [3–5]. While the Metabolic and Bariatric Surgery Accreditation and Quality Improvement Program recommends preoperative weight loss as a tool for optimizing comorbidities in certain patient populations, these recommendations are based on limited evidence suggesting that increased preoperative weight loss may result in a greater reduction in surgical complications [6]. For instance, it has been suggested that laparoscopic surgery may be more challenging in a patient living with obesity due to limited visualization of the abdomen and difficulty in manipulating surgical instruments. Patients with obesity also have a greater prevalence of enlarged fatty liver, which may contribute to increased conversions to open surgery [7, 8]. It has been suggested that weight loss assert that it may decrease liver size and lead to a reduction in surgical complications, operative time, and morbidity in patients undergoing MBS. Individual surgeons and programs may recommend preoperative weight loss based on the specific needs and circumstances of the patient [2]. Several studies on preoperative diets, such as meal replacement diets [9–17], intragastric balloons [18], pharmacologic therapy [19, 20], or home-based regimens [21], have reported decreases in body weight, visceral fat, and/or liver size. Greater preoperative weight loss was associated with a mild decrease in length of stay but was not associated with a reduction in operative time, overall complication rates, ICU admissions, or intraoperative complications. The inconclusive literature does not support the medical necessity of weight loss prior to bariatric surgery for reducing surgical complications or predicting successful postoperative weight loss. Therefore, this narrative review aimed to describe the current evidence on the effect of a noninvasive preoperative weight loss, by dietary or pharmacotherapy or space-occupying devices on preoperative weight loss, peri-operative complications, hospital length of stay, and post-operative complications in patients with obesity scheduled for MBS. We conducted a literature search and screening for relevant publications from January 2010 to June 2024.

## Materials and methods

**Fig. 1:**
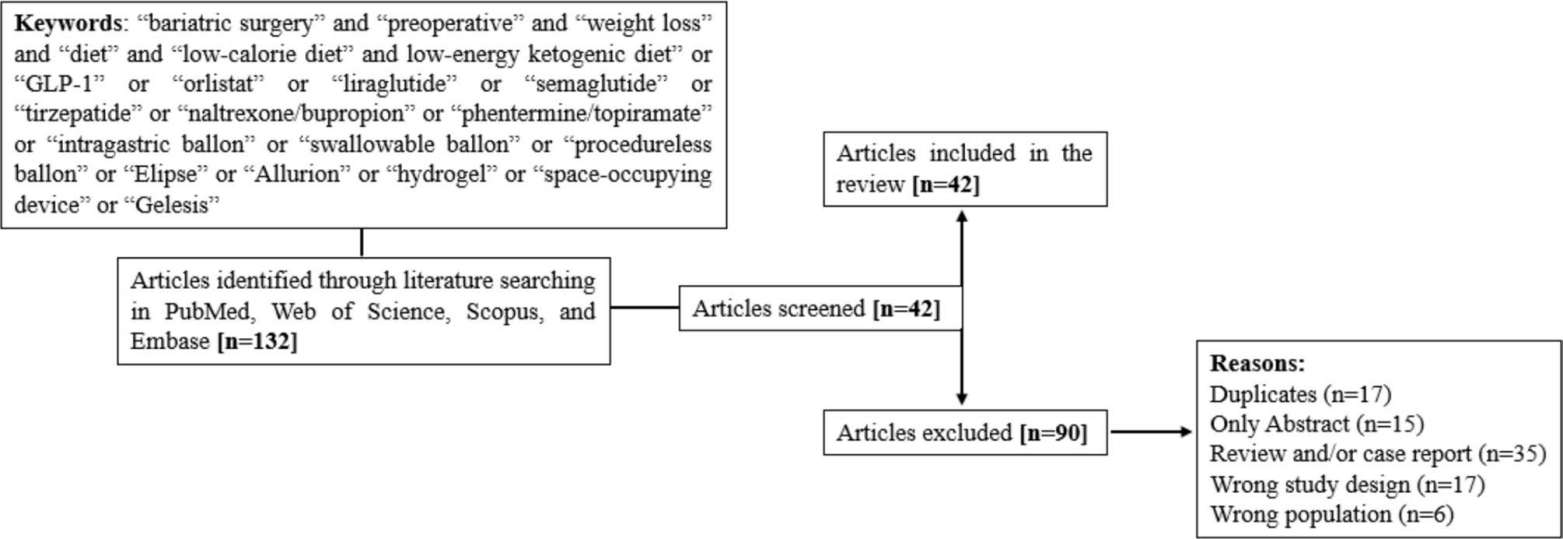
Flowchart of literature search and study selection. This figure illustrates the process of identifying and selecting studies included in the review. It outlines the number of records screened and excluded at each step and summarizes the final number of studies analyzed. The diagram follows PRISMA flowchart standards for transparency in systematic literature selection. *VLCD* very low-calorie diet, *VLEKT* very low-energy ketogenic therapy, *GLP-1 RA* glucagon-like peptide-1 receptor agonist, *IGB* intragastric balloon

We performed a search on PubMed, Web of Science, Scopus and EMBASE using the following keywords: “bariatric surgery" and "preoperative" and "weight loss" and “diet” or “low-calorie diet” or “low calorie ketogenic diet or “GLP-1” or “orlistat” or “liraglutide” or “semaglutide” or “tirzepatide” or “naltrexone/bupropion” or “phentermine/topiramate” or “intragastric balloon” or “swallowable balloon” or “procedureless balloon” or “Elipse” or “Allurion” or “hydrogel” or “space-occupying device” or “Gelesis”. Articles published from January 2010 to June 2024 were analyzed. Only English-language studies were included. Incomplete and non-peer reviewed preprint articles were excluded. We focused our attention on randomized controlled trials (RCTs), meta-analyses, systematic reviews, and observational cohort studies. The articles included were either prospective or retrospective, monocentric or multicenter studies, with a variable sample size. As shown in Fig. 1, from a total of 132 articles analyzed, 42 items were selected and 90 excluded.

## Results

The results of our review have been organized into paragraphs, divided into fields of strategies used for achieving preoperative weight loss through noninvasive approaches in patients with obesity scheduled for MBS.

### Preoperative weight loss before MBS: dietary approach

Before MBS, excessive body weight, increased liver volume (especially the liver left lateral section, LLLS) and liver steatosis may complicate the surgical procedure [22]. In particular, liver steatosis, showing a prevalence range between 52 and 90% in pattients with obesity scheduled for surgery, may complicate MBS when the LLLS is massively enlarged, limiting access to the esophagogastric junction and increasing the risk of laceration of the soft fatty liver with consequent bleeding [7]. In turn, these difficulties may result in an increased operative time, suboptimal surgery, and an increased rate of conversion to open surgery [7, 22].

To achieve moderate weight loss and reduction of liver volume and steatosis before MBS, several dietary protocols have been introduced over time, among which very low-calorie diets (VLCD) and very low energy ketogenic therapy (VLEKT) are widely prescribed in the last months before surgery [8, 12, 23]. VLCD regimens have already been investigated and have an established role in pre-MBS weight loss [24].

Schiavo et al. [21], with the aim to evaluate the clinical impact of a low-calorie diet on liver size, visceral fat, fat mass, and fat-free mass in patients undergoing sleeve gastrectomy demonstrated that an 8-week preoperative low-calorie diet is safe and effective in reducing body weight (− 16%, *p* < 0.05), liver size (− 16%, *p* < 0.05), visceral fat (− 10%, *p* < 0.05), and fat mass (− 10%, *p* < 0.05) with no significant reduction in fat-free mass (− 6%, *p* = 0.07). Furthermore, all tested patients showed high rates of acceptance and compliance in following the diet, and no side effects were observed.

In a recent systematic review and meta-analysis of randomized controlled trials on the clinical effects of VLCD prior to bariatric surgery, McKechnie et al. [25], found that 4 RCTs involving 294 participants who received liquid formula VLCDs preoperatively and 294 participants who received a non-VLCD control met the inclusion criteria. The results showed that patients receiving VLCDs experienced significantly greater preoperative weight loss than patients receiving a control (mean difference, MD 3.38 kg, 95% confidence interval, CI 1.06–5.70, *p* = 0.004, *I*^2^ = 95%). Furthermore, they also found a non-significant reduction in 30-day postoperative morbidity in patients who received VLCD before bariatric surgery (risk ratio, RR 0.67, 95% CI 0.39–1.17, *p* = 0.16, *I*^2^ = 0%), confirming that the impact of preoperative VLCDs on postoperative outcomes following bariatric surgery remains unclear.

In line with these findings, a recent randomized controlled study by Güçlü et al. [26] has provided novel insights into the epigenetic effects of preoperative dietary interventions. The authors demonstrated that adherence to a 2-week VLCD prior to MBS was associated with alterations in ghrelin DNA methylation levels, which could play a role in appetite regulation. These findings support the hypothesis that preoperative dietary regimens may have favorable effects not only on surgical outcomes but also on patient behavior, potentially improving hunger management and postoperative adherence through neuroendocrine modulation. The role of VLEKT is consolidated and increasing in importance for obesity treatment [27], but their role before MBS is still less considered. In addition, in the preoperative period, some issues have to be taken into consideration as the influence of the catabolic state and oxidative stress induced by LEKT. One of the first studies addressing the effect of VLEKT on patients with obesity scheduled for BS was performed by Leonetti et al. [28]. The study evaluated the efficacy of a sequential diet regimen called OPOD, in 50 patients with a mean BMI of 53.5 ± 8.4 kg/m^2^, with and without type 2 diabetes mellitus (T2DM) who were scheduled for laparoscopic MBS. The OPOD regimen consisted of a 10-day KD (600 kcal/day, 15 g of carbohydrates, 80 g of proteins, and 23 g of lipids), followed by a 10-day VLCD (800 kcal/day, 55 g carbohydrates, same proteins, and 30 g lipids), and finally an LCD (1100 kcal/day, with an increase in carbohydrates up to 145 g, 60 g proteins, and 33 g lipids) until the surgery. Participants were assessed at baseline (T0) and after 10 days (T1), 20 days (T2), and 30 days (T3). The results showed that body weight, BMI, waist circumference, and neck circumference were significantly lower at T1, T2, and T3 than at T0 in the 48 patients who completed the OPOD regimen. Additionally, in patients with T2DM, fasting plasma glucose levels decreased significantly, allowing for a reduction in diabetic medications. The study concluded that the OPOD, which includes 10 days of VLEKT, was safe and effective for patients with obesity with or without T2DM who were candidates for MBS. Schiavo et al. [8] demonstrated that a 4-week preoperative LEKT is safe and effective in reducing body weight (− 10.3%, *p* < 0.001, in men;—8.2%, *p* < 0.001, in women) and left hepatic lobe volume (− 19.8%, *p* < 0.001) in patients living with obesity scheduled for MBS. Furthermore, Pilone et al. [17] proposed a dedicated KetoStationkit for use during the first 10 days of the regimen, along with a hypocaloric diet for the next 20 days. The KetoStationkit included protein powder (82 g of protein from whey and caseinate for every 100 g of product) and nutritional supplements (multiminerals, multivitamins, and omega-3 fatty acids). Participants were advised to consume eight scoops of ketogenic powder per day for females and nine scoops per day for males, with each scoop diluted in 100–200 mL of water (one scoop containing 10 g, including 0.3 g of carbohydrate, 8.2 g of protein, and 0.4 g of fat). Patients could add vegetables to their regimen during lunch and dinner and were encouraged to consume at least 2 L of fluids per day. Ketone body levels were measured in the plasma and urine, and routine laboratory tests and anthropometric measurements were conducted at enrolment (T0), after 10 days (T1), and after 30 days (T2). The results of the study showed a significant decrease in body weight, BMI, and waist circumference at T0 and T1, T0 and T2, and T1 and T2 (*p* < 0.05). A bioelectrical impedance assay showed a significant reduction in visceral fat at T1 and T2. The study also observed a significant improvement in several clinical parameters, including glycemic and lipid profile parameters, associated with a mean 30% reduction in liver volume. The authors concluded that a VLEKT performed using a dedicated KetoStationkit was safe and effective in reducing weight and liver volume in patients with obesity who were candidates for MBS [13].

Concerning the mechanisms through which LCDs and LEKDs impact surgical outcomes, they exert their effects primarily through rapid mobilization of hepatic and visceral fat stores. Caloric restriction leads to a negative energy balance, promoting lipolysis and reducing glycogen stores, which are associated with decreased hepatic water content and overall liver volume [29, 30]. In LEKT, the shift from glucose to ketone metabolism enhances fat oxidation, further accelerating the depletion of intrahepatic and visceral adipose tissue [30]. These metabolic adaptations result in a measurable decrease in liver size and visceral fat mass, thereby improving surgical exposure and reducing intraoperative complexity [29, 30]. Additionally, weight loss induced by these dietary interventions improves insulin sensitivity by reducing ectopic fat deposition in insulin-responsive tissues, including the liver and skeletal muscle [25, 31]. This contributes to better glycemic control and a reduction in circulating pro-inflammatory cytokines such as TNF-α and IL-6, ultimately lowering perioperative complication rates and supporting more favorable postoperative recovery trajectories [29, 32].These mechanisms underscore the importance of dietary optimization as a foundational element of preoperative care in MBS.

In addition, Albanese et al. [23], aiming to compare surgical outcomes and weight loss in two groups of patients who received two different types of preoperative diet (LCD and LEKT), reported that ketogenic diets had better impact than low-calorie diets on surgical outcomes, influencing drainage output, postoperative hemoglobin levels, and hospital stay.

Furthermore, to macronutrient-focused dietary interventions, it is essential to address potential micronutrient deficiencies before surgery. Tang et al. [33] reported a high prevalence of deficiencies in key vitamins and minerals, including vitamin D, vitamin B12, folate, iron, and zinc, among patients with obesity scheduled for MBS. These deficiencies, if uncorrected, may predispose individuals to increased perioperative complications and delayed recovery. Therefore, preoperative nutritional protocols should systematically include micronutrient screening and appropriate supplementation. This recommendation is supported by Khalooeifard et al. [34], whose meta-analysis emphasized that correcting micronutrient deficiencies as part of a structured preoperative regimen contributes to better surgical outcomes and reduced complication rates.

In conclusion, weight loss before MBS is crucial for patients, as it leads to various benefits, such as a decrease in liver volume and visceral fat, a lower risk of intra- and post-operative complications, shorter surgery times, and reduced hospital stays. The impact of preoperative VLCDs on postoperative outcomes following MBS remains unclear. It is possible that VLCDs may contribute to decreased postoperative morbidity, but further larger prospective trials are required to investigate the signal identified in this study. Furthermore, large trials investigating the use of preoperative VLCDs in patients with obesity undergoing MBS are required to assess the generalizability of these results, in order to optimize the management of a steadily growing patient population. VLEKTs have proven to be a safe and effective way to achieve weight loss and may be considered an option in the preoperative period of MBS. However, larger RCTs with well-defined dietary protocols are necessary to make definitive conclusions. A summary of the articles describing the role of pre-bariatric diet-induced weight loss is reported in Table 1.

**Table 1:** A summary of the articles describing the role of pre-bariatric diet-induced weight loss

Author, year	Study design	N	Surgical procedure (SP)	Dietetic intervention	Duration of intervention	Pre-op weight loss/other side effects	Peri-op complications	Length of stay	Post-op complications	Post-op weight loss/other side effects
Iannelli et al. 2022	RCT	47	RYGB/SG	Omega-3 supplements	4 weeks	LSR:	NR	NR	NR	NR
Van Wissen J et al. 2016	Systematic Review	281	All SP	VLCD, nutritional supplements,BIB	4–12 weeks	LSR: VLCD 14%; nutritional supplements 20–43%, BIB 32%	NR	NR	NR	NR
Leonetti F et al., 2015	Prospective study	50	SG	VLEKT + VLCD + LCD (OPOD diet) vs LCD	30 days	− 13 kg average in OPOD group; − 7 kg in control group	NR	NR	NR	NR
Lorenzo PM et al., 2022	RCT	111	All SP	VLEKT/LCD	4–6 months	− 12.4% BMI reduction in VLEKT; more reduction of inflammatory cytokines in VLEKT	NR	NR	NR	NR
Schiavo L et al., 2021	RCT	48	IGB	VLEKT/VLCD		> FM reduction in VLEKT group despite FFM	NR	NR	NR	> WL and FFM reduction in LCD group
Andrianzen Vargas M et al., 2011	Review	371	IGB	LCD/VLCD	2 weeks to 6 months	VLEKT 15% WL at 12 weeks in all patients’ vs IGB 12% WL at 6 months	NR	NR	NR	NR
Schiavo L et al., 2015	Prospective study	37	SG	Mediterranean Diet	8-weeks	− 16% BMI; − 29% LLLS; − 7% FFM	NR	NR	NR	NR
McKechnie T et al., 2023	Review	588	RYGB/SG	VLCD	2–4 weeks	− 6.2 kg WL vs − 3.4 kg in control group	NR	NR	Not significant	NR
Barrea L et al., 2023	Review	492	RYGB/SG	VLEKT	2–4 weeks	Weight loss, LLLS reduction, waist circumference reduction and other metabolic parameters	Reduction	Reduction	Reduction	NR
Schiavo L et al., 2018	Prospective study	27	All SP	VLEKT	4 weeks	− 10% WL, − 19.8% LLLS reduction	NR	NR	NR	NR
Pilone V et al., 2018	Prospective study	119	All SP	VLEKT	1 month	− 10% WL, 30% mean reduction in liver volume, significative reduction of metabolic parameters	NR	NR	NR	NR
Albanese A et al., 2019	RCT	178	SG	VLEKT/VLCD	1 year	− 5.8 kg WL in VLEKT	NR	Slight reduction in VLEKT	Reduction in VLEKT	NR

*LLS* liver left lobe size, *FFM* fat free mass, *FM* fat mass, *LEKT* very low energy ketogenic therapy, *VLCD* very low calorie diet, *LCD* low calorie diet, *BMI* body mass index, *IGB* intragastric balloon, RYGB

### Preoperative weight loss before MBS: pharmacotherapy approach

Obesity management medications (OMMs) have emerged as a critical component in managing obesity, both as stand-alone treatments and in conjunction with MBS. Currently available OMMs include orlistat, phentermine/topiramate (not approved in the European Union), naltrexone/bupropion, and glucagon-like peptide-1 receptor agonists (GLP-1RAs) such as liraglutide and semaglutide. The latter have been shown to reduce body weight by 8–15%. Newer GLP-1-based agents like tirzepatide can lead to an average weight loss of 21% [35]. Other OMMs, such as orlistat, phentermine/topiramate, and bupropion/naltrexone, also offer varying degrees of weight loss, albeit with different mechanisms of action and side effect profiles [36].

The impact of pharmacotherapy on outcomes before and after bariatric surgery remains unclear. Many people with obesity and associated complications, such as diabetes, require pharmacological therapy to manage their disease perioperatively and postoperatively. The use of pharmacotherapy in such individuals can affect different bariatric surgery outcomes, such as weight loss, glycemic control, and the development of postoperative complications [37]. Thus, the use of pharmacotherapy in the preoperative setting is gaining attention, as it may help reduce surgical risks by facilitating preoperative weight loss, although it is still underutilized [19, 20]. Specific indications regarding the preoperative BMI cut-off for initiating weight management programs are lacking, a wide range of baseline BMIs is reported in the literature and there are no criteria for the clinical selection of individuals who would benefit from pre-surgical therapy. Standardized terminology is also lacking. A recent scoping review proposed adopting the term “neoadjuvant” to describe preoperative OMM use and “adjuvant” for postoperative use, highlighting the need for consistency in future research [38]. These studies examined the use of OMMs like orlistat, GLP-1RAs, and phentermine/topiramate in patients undergoing different bariatric procedures, including sleeve gastrectomy (SG), Roux-en-Y gastric bypass (RYGB), and gastric banding (Table 2).

**Table 2:** A summary of the articles describing the role of pre-bariatric pharmacotherapy-induced weight loss

Author, year	Study design	N	Surgical procedure	AOM	AOM duration	Pre-op weight loss	Peri-op complications	Length of stay	Post-op complications	Post-op weight loss
Malone et al., 2012	Randomized controlled trial	38	Gastric bypass	Orlistat	6 months	3.6% EW at 6 months	NR	NR	NR	NR
Ard et al., 2019	Prospective	13	Sleeve gastrectomy	Phentermine/Topiramate	3–6 months	11.2% TBWL at 2-years	NR	NR	Blood pressure reduction	11.2% TBWL at 2-years
Stier et al., 2022	Proof of concept study	26	Bariatric surgery (unspecified)	Liraglutide	20.7 ± 6.9 days	27.5 kg average	NR	NR	NR	NR
Ilang et al., 2023	Retrospective cohort study	31	Gastric bypass, sleeve gastrectomy	GLP-1 agonist (semaglutide, liraglutide, dulaglutide)	4.9 months	5.5-point BMI reduction vs. 2.9 in controls	3 reported in GLP-1 group	NR	3 in GLP-1 group	2.5-point BMI reduction
Martines et al., 2023	Retrospective study	86	Laparoscopic sleeve gastrectomy	Liraglutide vs. IGB	NR	Significant BMI reduction (IGB)	No difference between groups	NR	No difference between groups	IGB group > liraglutide
Cunningham et al., 2023	Retrospective chart review	98	Sleeve gastrectomy, Roux-en-Y	Phentermine/Topiramate	NR	31.3% TBW	NR	NR	NR	25.3% TBW
Rubio-Herrera et al., 2023	Retrospective observational study	102	Bariatric surgery (unspecified)	Liraglutide, Semaglutide	52 weeks	16.9% with semaglutide, 16.1% liraglutide	NR	NR	NR	NR
Lu et al., 2023	Retrospective study	55	One-anastomosis gastric bypass	Orlistat	10–14 days	3.1% EWL, 1.7% TWL (Respondents)	None reported	No difference (3 days median)	No difference at 30 days	No significant difference at 2 years
Muñoz et al., 2024	Quasi-experimental prospective study	37	Bariatric-metabolic surgery	Liraglutide	12 weeks	5.5% at 3 months	NR	NR	NR	NR

*AOM* anti-obesity medication, *IGB* intra-gastric balloon, *NR* not reported

In a randomized controlled trial, Maloneet al. [39] evaluated the use of orlistat, a lipase inhibitor, in people undergoing RYGB. Orlistat was used as an adjunct therapy to help people achieve a 10% preoperative weight loss. The study included 38 participants (19 in the treatment group and 19 controls). At 6 months, the orlistat group achieved a 3.6% excess weight loss (EWL), significantly lower than the control group’s 10.2% EWL. The orlistat group had a total body weight loss (TBWL) of 2.0% at 6 months compared to the control group's 5.4%. Overall, the findings suggested that orlistat offered limited benefit in the preoperative management of weight loss, with some patients reporting gastrointestinal side effects that impacted adherence. Loet al*.* [40] investigated the use of a preoperative short-term orlistat-based regimen in 55 Asian individuals undergoing one-anastomosis gastric bypass (OAGB). Despite all patients following the same preoperative protocol, including orlistat 120 mg daily, there was variability in weight outcomes: some patients lost weight (EWL 3.1%, TBWL 1.7%), while others gained weight (an increase of 4.9% EWL and 2.2% TBWL) preoperatively. Those who lost weight had a numerically shorter operation time (107 min vs. 140 min, p = ns). There were no significant differences between the groups in terms of long-term outcomes, including weight loss at 2 years postoperatively.

Ard et al*.* [41] conducted a prospective long-term study on the effects of phentermine/topiramate on weight loss and cardiovascular outcomes. Thirteen individuals with a BMI ≥ 50 kg/m^2^ on a waiting list for SG were treated with phentermine/topiramate for 3–6 months before surgery. Patients achieved 11.2% greater TBWL 2 years after surgery compared to controls who underwent SG alone. Furthermore, the combination of phentermine/topiramate and surgery resulted in greater improvements in blood pressure, suggesting that combining SG with extended-release phentermine/topiramate may be a viable option for patients with severe obesity. *Cunningham *et al. [42] conducted a retrospective chart review to evaluate the use of phentermine/topiramate in patients with a BMI over 60 kg/m^2^ undergoing SG or RYGB. A total of 98 participants were included, 8 of whom were treated preoperatively. Participants treated with phentermine/topiramate preoperatively achieved a 31.3% TBW loss in 1 year, significantly greater than patients who received pharmacotherapy in the first postoperative year (25.3% TBWL) or no pharmacotherapy (20.8% TBWL). Stieret al. [43] conducted a proof-of-concept study exploring the use of a fast-track rescue weight reduction (RWR) therapy in patients with acutely life-threatening severe obesity (BMI ≥ 60 kg/m^2^). The study involved 26 participants who were treated with liraglutide (initiated at 1.2 mg/day and increased to 1.8 mg/day over 3 days) combined with a leucine-rich amino acid infusion (320 kcal/day) and a hypocaloric diet (1000 kcal/day). The goal was to achieve rapid preoperative weight loss to enable surgery. Patients experienced an average weight loss of 27.5 kg within approximately 21 days. This rapid reduction allowed all individuals to proceed with surgery. The study highlighted liraglutide's potential for achieving significant weight loss in a brief time frame, making it a valuable tool in emergency preoperative settings. Perioperative complications were not significantly increased, and no major postoperative complications were reported. These data may be considered promising as part of a pharmaceutical fast-track bridging therapy to surgery, but it should be noted that a dose escalation period and at least 3–6 months at the therapeutic dose of liraglutide or other GLP1-RAs are recommended and generally needed to obtain a clinically relevant weight loss [44].

In a retrospective cohort study, Ilanget al. [45] investigated the use of GLP-1RAs in people with a BMI greater than 50 kg/m^2^ preparing for bariatric surgery. The study included 31 participants, 18 of whom received preoperative GLP-1RA (89% semaglutide, 5.5% liraglutide, 5.5% dulaglutide). Participants in the GLP-1RA group had a significantly greater preoperative weight loss (approximately 9.5% and 5.5 BMI-point reduction) compared to controls (approximately 5.2% and 2.9 BMI-point reduction, *p* = 0.026) over a period of 5–6 months. Importantly, there were no differences in perioperative complications between the two groups, and no complications related to the use of GLP-1RAs were reported. Martineset al. [46] compared the efficacy of intragastric balloon (IGB) therapy with liraglutide in individuals with severe obesity (BMI ≥ 50 kg/m^2^) preparing for laparoscopic SG. In this retrospective study, 44 individuals received IGB, and 42 were treated with liraglutide for 6 months. The IGB group achieved greater preoperative median percent EWL (15.5, IQR 13–18.7 vs. 6.71, IQR 5.8–7.4; *p* < 0.05) and median percent TBWL (28.5, IQR 24.8–33.07 vs. 11.8, IQR 10.3–14.3; *p* < 0.05) when compared to the liraglutide group. Postoperative weight loss was also greater in the IGB group at 6 and 12 months. Both treatments were well-tolerated, with no significant differences in postoperative complications between the groups. In an observational study, Rubio-Herreraet al. [47] evaluated the impact of GLP-1RAs (liraglutide 3.0 mg and semaglutide 1.0 mg) on preoperative weight loss in individuals on a waiting list for bariatric surgery. The study followed 102 participants treated with GLP-1RAs for at least 6 months. At the end of 52 weeks, those treated with semaglutide lost an average of 16.9% of their initial weight, and those treated with liraglutide lost 16.1%. Notably, 68.6% of participants were satisfied with their weight loss and withdrew from the waiting list for surgery. Finally, *Muñoz *et al. [48] conducted a quasi-experimental prospective study to assess the effectiveness of liraglutide as a preoperative weight loss therapy in individuals with severe obesity (BMI ≥ 48 kg/m^2^) preparing for MBS. A total of 37 participants were treated with liraglutide 3.0 mg for 12 weeks, achieving an average total weight loss of 5.5%. The study noted a high adherence rate, with 94.6% of patients achieving some degree of weight loss. No perioperative complications were directly attributed to liraglutide use. However, in another study carried out in 117 individuals with prior use of liraglutide and 101 controls, the incidence of adhesions was 22.2% in those undergoing SG on prior liraglutide intake whereas no adhesions were found in the control group [49]. While OMMs show potential for promoting preoperative weight loss before bariatric surgery, the evidence supporting their impact on postoperative outcomes remains limited. Most studies have small sample sizes and retrospective designs, and few have evaluated the effect of OMM-induced weight loss on perioperative or long-term postoperative outcomes. Larger, well-designed randomized trials are needed to establish optimal treatment protocols and assess their true benefits. In conclusion, OMMs remain a promising tool, particularly for high-risk patients, but further research is essential to confirm their role in MBS.

### Preoperative weight loss before MBS: space-occupying devices

Non-surgical weight-loss strategies include more- or less-invasive endoscopic procedures and devices aiming to restrict gastric volume and improve satiety, reducing food intake [50]. A novel generation of space-occupying devices are emerging as novel solutions to achieve weight loss avoiding both endoscopy and sedation, therefore reducing costs and complications related to the procedure [51]. Bridge therapies to induce preoperative weight loss have been used for patients with very severe obesity, defined as having a BMI $\ge$ 50 kg/m^2^. In this field, previous liquid or gas-filled intragastric balloon (IGBs) required endoscopy (ReShape, Spatz, LexBal, Orbera and Obalon or HelioSphere) at least for removal when swallowable, or for both placement and removal. Multiple IGB models are in use worldwide, each with a different safety and efficacy profile with different complication rate. A survey collected an experience of 20,680 IGBs of 12 different models, positioned in 21 spanish hospitals, showing a mean percentage total body weight loss (%TBWL) of 17.66 ± 2.5% with an early removal rate due to intolerance of 3.62%, major and minor complications rate of 0.70% (mainly gastric ulcer) and 6.37% (mainly esophagitis) [52]. To date, evidence of “procedureless” systems to achieve weight loss without or before surgery are emerging for the “Elipse” balloon and for ingestible, expandable, and biodegradable capsules such as “Gelesis” or “Hydrogel" or “Epitomee”. These devices can be swallowed and excreted without invasive procedures with low complication rate. However, literature on presurgical weight loss using these devices is still scarce, therefore the studies presented here are weight loss interventions without subsequent surgery. The “Elipse” balloon is a swallowable device filled with 550 mL of fluid (distilled water with potassium sorbate preservative) through a delivery catheter. The catheter is removed after balloon insertion and X-ray confirmation of correct positioning, without any sedation or endoscopy.

The balloon placement procedure takes approximately 20 min and is designed for a 16-weeks residence time. After this period, a degradable release valve spontaneously opens, the balloon empties, deflates, and is excreted naturally in the stool. Symptoms after ingestion can be managed with medications (antiemetics, anti-spasmodics, and proton pump inhibitors) and are self-limiting after a few days. Adverse events have a very low rate, and include early deflation, gastrointestinal obstruction, need for removal by endoscopy or surgery, early removal for intolerance or emesis, and very rarely gastric dilation or perforation, esophagitis or pancreatitis.

Among the studies on Elipse weight loss outcomes, safety and efficacy profile, Ienca et al., in a multicenter trial, reported a sample of 1770 participants achieving a %TBWL of 14.2 ± 5% after balloon deflation, with very low rate of early removal or major and minor adverse events [53]. Table 3 shows a collection of 17 trials on Elipse, mainly reporting 4-month interventions with or without a nutritional counseling, with follow-ups extending up to 8 months after balloon deflation. The reported %TBWL varied from Tønnesen’s 0.8% [54] to Patino Araujo’s 14.7% after 1 year of follow-up [55]. Notably, when the balloon intervention was combined with lifestyle coaching, as shown by Jense et al. [56], the mean weight loss after 1 year was 11 ± 8.4% or 12.6 ± 6.5% using multiple sequential balloons. This figure increased to 13.8 ± 7.6% when OMMs were combined with IGB. Moreover, Schiavo et al. investigated the effects of a standard LCD versus a LEKT, implemented during the 4-month Elipse intervention. The LCD and LEKT groups achieved 21% and 18% TBL, respectively. Interestingly, although the VLCD group had slightly lower weight loss than the LCD group, they experienced higher fat-mass loss and a lower decrease in fat-free mass and resting metabolic rate, making the quality of the diet an important concern during caloric restriction [18].

**Table 3:** A summary of the articles describing the outcomes of intervention including swallowable balloon (Elipse)

Author, year	Study design	N (M/F)	Intervention	Duration (months)	Weight loss		ΔBMI (kg/m2)	ΔWC (cm)		Metabolic improvements	AE
Machytka et al., 2017	Prospective, observational, open-label	34 (11/23)	IGB	4	10 ± 6.6%TBWL		3.9 ± 3.1	8.4 ± 6.5		BP, TG, LDL, HbA1c,	1 aborted
Raftopoulos et al., 2017	Prospective non-RT	11 (5/7)	IGB (with counseling) + FU	4 + 8	15.4 kg %EWL: 50.2 (4 m); 17.6 (12 m) %TBWL: 14.6 (4 m); 5.9 (12 m)		5.4	NR		Diastolic BP, HbA1c, Cholesterol, TSH, liver transaminase	none
Al-Subaie et al., 2017	Prospective pilot study	51 (4/47)	IGB		8.84 kg 10.44%TBWL 40.84%EWL		3.42	8.62			5 intolerance 1 IGB vomited 1 ED
Alsabah et al., 2018	Multicenter prospective	135	IGB	4	13.12 ± 6.1 kg 15.1 ± 9.5%TBWL		4.9 $\pm$ 2.2	NR			2 IGB vomited, 3 intolerance, 3 ED, 1obstruction At deflation: 18 diarrheas 29 abdominal pain
Genco et al., 2018	Prospective	38 (10/28)	IGB	4	12.7 kg 26%EWL 11.6%TBWL		4.2	12.5		BP, TG, Glucose, HOMA-IR	1 endoscopical removal
Jamal et al., 2019	Prospective Non-RT	112	IGB + FU	4 + 8	%TBWL: 10.7 (3 m) − 10.9 (6 m) − 7.9 (12 m)						6 intolerance 1 obstruction, 3 ED
Espinet Coll et al., 2019	Prospective Non-RT	30 (28/2)	IGB	4	11.2 kg 12.1 $\pm$ 5.8%TBWL 64.7 ± 25%EWL		4.1	NR			2 ED, 1 IGB vomited, 1 obstruction, 1 intolerance
Ienca et al., 2020	Multicenter prospective non-RT	1770 (506/1264)	IGB	4	13.5 ± 5.8 kg 67 ± 64.1%EWL 14.2 ± 5%TBWL	4.9 ± 2				HbA1c, LDL, TG	51 intolerance, 11 ED, 3obstruction 4 hyperinflation, 6 other
Schiavo et al., 2021	Pilot, prospective, RCT	48 (22/26)	IGB (LCD *vs* LEKT)	4	LEKT 20.2 kg LCD 22.4 kg LEKT 18%TBWL LCD 21%TBWL		NR		NR	Cholesterol tot., LDL, HDL, insulin and glucose, liver transaminase	none
Taha et al., 2021	Retrospective	96 (28/68)	IGB	4	11.2 ± 5.1 kg 12.1 ± 5.2%TBWL		4.9 $\pm$ 2		10.9 $\pm$ 2.1	HbA1c, LDL, TG	3 intolerance, 1 ED At deflation: 3 IGB vomited, 11 diarrhea, 21 abdom.pain
TØnnesen et al., 2022	2 center pilot study	19 (14/5)	IGB (with diet) + FU	4 + 8	%TBWL: 3.9 (4 m); 0.8 (12 m)		NR		NR		1 emesis 1 obstruction
Jense et al., 2023	Retrospective	336 (95/241)	IGB + FU (with coaching) IGB + OMM Sequential IGB	4 + 8	%TBWL: 10 ± 4 (3 m)–11.83 ± 5.99 (6 m)–11 ± 8.4 (12 m) %TBWL: 13 ± 3.8 (3 m)–14.2 ± 5.1 (6 m)–13.8 ± 7.6 (12 m) %TBWL: 10 ± 3.6 (3 m)–11.5 ± 4.9 (6 m)–12.6 ± 6.5 (12 m)		NR		NR		7 ED 1 obstruction
Mathur et al., 2024	RCT	108 (60/48) 53 (29/24) 55 (31/24)	IGB + AOM* IGB	4	%TBWL (1–2–3–4 m): 7.9–12.5–15.2–17.6 6.1–10.5–12.8–13.7		NR		NR	HbA1c, glucose, BP, Cholesterol Tot., LDL, HDL, TG	3 + 1 intolerance
Kosai et al., 2024	Prospective	486	IGB + FU	4 + 8	9.6 (4 m)–12.8 (12 m) kg %TBWL: 10.5 (4 m)–13.7 (12 m)		3.8 (4 m) – 5 (12 m)		NR		1 hyperinflation
Sacher et al., 2024		107 (11/96)	IGB + FU (with coaching)	4 + 2 + 6 (12 total)	%TBWL: 10.9 (4 m)–13.5 (6 m)–11.22 (12 m)						
Patino Araujo et al., 2024		167		4 + 8	%TBWL: 15.7 ± 5.2 (4 m)–17.1 ± 6 (6 m)–14.7 ± 18 (12 m) EWL%: 60.1 ± 29.3 (4 m)–65.3 ± 34% (6 m)–43.1 ± 64.6 (12 m)						4 intolerance
Dejeu et al., 2024	Retrospective, observational, single-center	571		4	13.9 kg		4.4				4 intolerance

*RT* randomized trial, *RCT* randomized controlled trial, *IGB* intra-gastric balloon, *FU* follow-up, *EWL* excess weight loss, *TBWL* total body weight loss, *WC* waist circumference, *BP* blood pressure, *TG* triglycerides, *HbA1c* glycated hemoglobin, *AE* adverse events, *LCD* low calorie diet, *LEKT* low energy ketogenic therapy, *OM* obesity management medication, *NR* not reported, *ED* early deflation

Another minimally invasive device to reduce gastric volume by occupying space is represented by superabsorbent polymers that self-expand in the stomach, forming a gel structure that mimicks the ingestion of solid food. This dry polymer technology can absorb up to 1L/g of fluid, transforming powder granules into a 3D hydrogel structure that mixes with meals, increasing volume without extra caloric intake. Adverse events reported include abdominal discomfort, bloating, nausea and constipation. Safety and efficacy studies on these procedureless devices have been published for weight loss. To date, no presurgical studies are available to evaluate their perioperative and postoperative outcomes. Therefore, Table 4 shows the main weight-loss outcomesreported in the four available studies on hydrogel capsules [57–60]. The rate of weight loss is not comparable to IGBs. Moreover, the sample size, quality of the reports, and length of these interventions are not yet sufficient to draw conclusions on their efficacy and safety in the presurgical setting.

**Table 4:** A summary of the articles describing the outcomes of intervention including hydrogel capsules

Author, year	Study design	N (M/F)	Intervention	Duration (months)	Weight loss	ΔBMI (Kg/m2)	ΔWC (cm)	Metabolic improvements	AE
Shirin et al., 2019	Prospective	78 (58/20)	Epitomee device + diet	3	3.2 $\pm$ 2.7 kg 3.7 $\pm$ 3%TBWL	1.2 $\pm$ 1	3.7 $\pm$ 3.7		None
Greenway et al., 2019	Multicenter RCT	223(98/125) *vs* 213(93/120)	Gelesis100 *vs* placebo	6	6.4 vs 4.4%TBWL 29 vs 21%EWL	2.1 *vs* 1.5	6.7 *vs* 5	BP, LDL, HOMA-IR	Gastrointestinal-related
Reister et al., 2022	Double-blind RCT cross-over	18 (5/13) + 10 (5/5)	Gelesis capsules + Meal-provided *or* free-meal	1 week	$0.53\pm$ 0.2 kg 0.06 $\pm$ 0.31 kg		NR		
Shirin et al., 2023	Prospective non-RT	78 (58/20)	Epitomee device + counseling	3	− 4.0 ± 2.6 kg 4.5%TBWL			BP	None

*RCT* randomized controlled trial, *EWL* excess weight loss, *TBWL* total body weight loss, *WC* waist circumference, *BP* blood pressure, *TG* triglys, *AE* adverse events, *NR* not reported

## Strengths, limitations, conclusions, and future directions

**Fig. 2:**
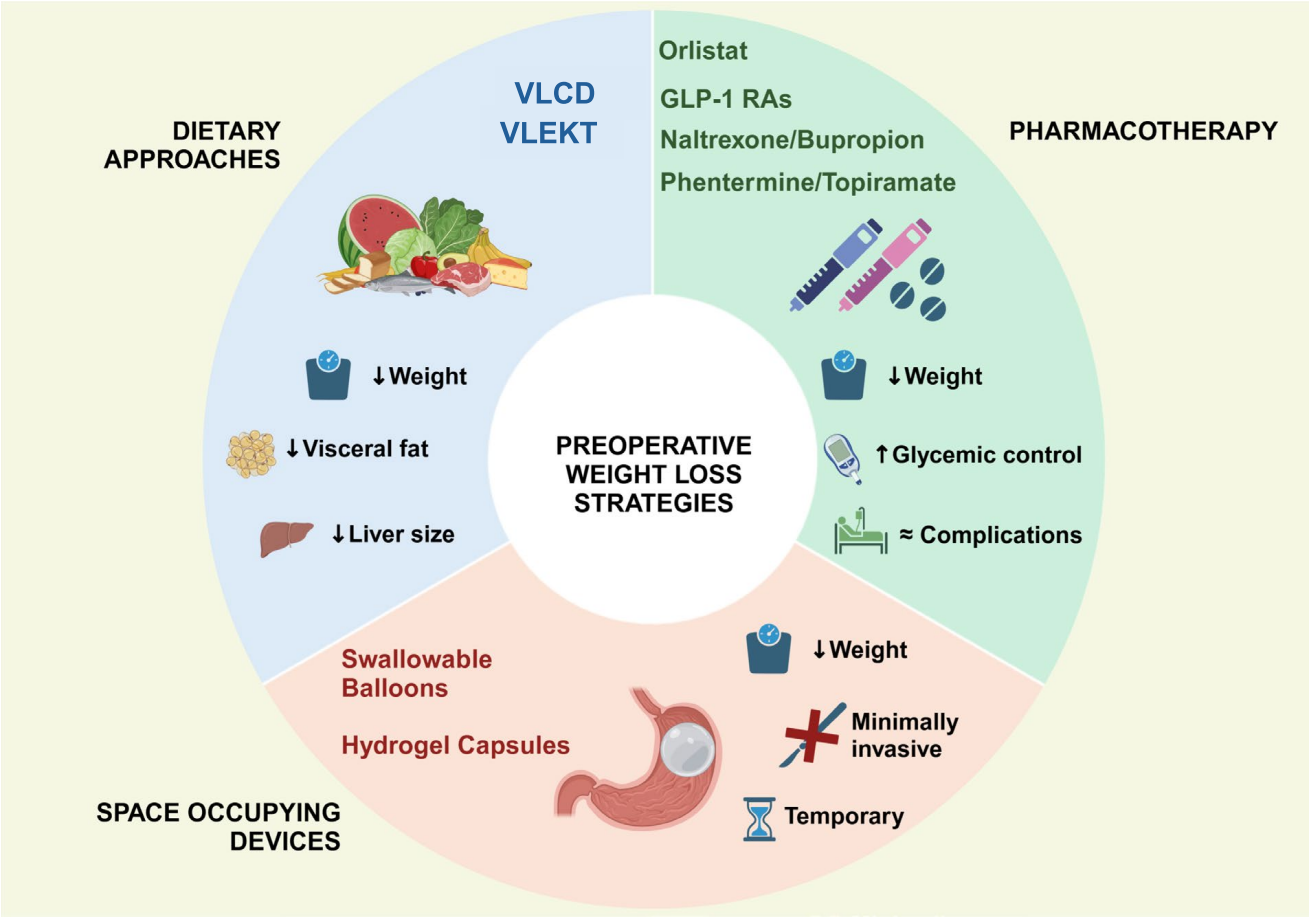
Summary of outcomes across different preoperative weight loss interventions: this schematic overview illustrates the key clinical outcomes associated with various noninvasive strategies for preoperative weight loss in patients undergoing metabolic and bariatric surgery (MBS). Interventions include dietary approaches as very-low calorie diet (VLCD) and very low energy ketogenic therapy (VLEKT), pharmacological agents, as glucagon-like peptide 1 receptor agonists (GLP-1 RAs), orlistat, naltrexone/bupropion), and space-occupying devices (e.g., swallowable balloons and hydrogel capsules). **Symbols:** ↓ = Reduction; ↑ = Improvement; ≈ = Mild or limited complications; X = Surgery not required;  = Short-term intervention

This review highlights the emerging role of noninvasive, short-term weight loss strategies, including dietary interventions, pharmacological agents, and space-occupying devices, as valuable tools in the preoperative optimization of candidates for BMS. These approaches contribute to reductions in liver volume and visceral adiposity, improvements in glycemic and inflammatory profiles, and in selected cases, early weight loss, all of which may reduce perioperative risks and enhance surgical feasibility (Fig. 2).

Among the strengths of this review are its comprehensive scope, the inclusion of recent evidence across multiple intervention types, and the structured comparison of clinical outcomes. In addition, this is one of the few reviews to provide a comparative summary of diverse noninvasive preoperative strategies, presented in a manner accessible to multidisciplinary teams.

Nevertheless, several limitations in the current literature must be acknowledged. Most available studies are limited by small sample sizes, heterogeneous designs, short intervention periods, and a lack of standardized endpoints. There is notable variability in how outcomes are reported (e.g., %TBWL vs. %EWL, liver volume, BMI), making cross-study comparisons difficult. Furthermore, key patient-related variables, such as adherence, baseline nutritional status, and comorbidities, are often underreported or insufficiently controlled for, reducing the external validity of findings.

Future research should prioritize well-designed, multicenter randomized controlled trials with harmonized outcome measures and long-term follow-up to assess the durability and broader impact of these strategies. Further exploration of patient-centered outcomes, cost-effectiveness, and integration into multidisciplinary care pathways will also be critical to inform clinical guidelines and support personalized preoperative management in bariatric surgery.

## Data Availability

Not applicable.
